# Blood flow-restricted exercise in space

**DOI:** 10.1186/2046-7648-1-12

**Published:** 2012-12-01

**Authors:** Kyle J Hackney, Meghan Everett, Jessica M Scott, Lori Ploutz-Snyder

**Affiliations:** 1 Wyle Science, Technology and Engineering Group, Houston, TX 77002, USA; 2 University of Houston, Houston, TX, 77002, USA; 3 Universities Space Research Association, Houston, TX, 77002, USA

**Keywords:** Blood flow-restricted exercise, KAATSU, Microgravity, Spaceflight, Bed rest, Unloading

## Abstract

Prolonged exposure to microgravity results in chronic physiological adaptations including skeletal muscle atrophy, cardiovascular deconditioning, and bone demineralization. To attenuate the negative consequences of weightlessness during spaceflight missions, crewmembers perform moderate- to high-load resistance exercise in conjunction with aerobic (cycle and treadmill) exercise. Recent evidence from ground-based studies suggests that low-load blood flow-restricted (BFR) resistance exercise training can increase skeletal muscle size, strength, and endurance when performed in a variety of ambulatory populations. This training methodology couples a remarkably low exercise training load (approximately 20%–50% one repetition maximum (1RM)) with an inflated external cuff (width, ranging between approximately 30–90 mm; pressure, ranging between approximately 100–250 mmHg) that is placed around the exercising limb. BFR aerobic (walking and cycling) exercise training methods have also recently emerged in an attempt to enhance cardiovascular endurance and functional task performance while incorporating minimal exercise intensity. Although both forms of BFR exercise training have direct implications for individuals with sarcopenia and dynapenia, the application of BFR exercise training during exposure to microgravity to prevent deconditioning remains controversial. The aim of this review is to present an overview of BFR exercise training and discuss the potential usefulness of this method as an adjunct exercise countermeasure during prolonged spaceflight. The work will specifically emphasize ambulatory BFR exercise training adaptations, mechanisms, and safety and will provide directions for future research.

## Review

### Introduction

Acute microgravity exposure results in rapid cephalad fluid shifts, space motion sickness, vestibular impairment, and musculoskeletal unloading [1]. However, the overall space environment encompasses both microgravity exposure and related challenges such as increased background radiation, social isolation, disruption of circadian rhythm (sunrise every 90 min), and access to a limited food variety (high salt prepackaged) and water supply [1]. These environmental stimuli interact to elicit chronic physiological adaptations including decreased bone mineral content and density, compromised maximal aerobic capacity, and reduced skeletal muscle mass and strength [2]. The morphological and structural alterations that occur within the skeletal muscle tissue as a result of microgravity exposure were uncovered following several Shuttle Transport System, Mir, and International Space Station (ISS) investigations [3-5]. These data suggest that the rate of skeletal muscle loss relative to the duration of microgravity exposure is nonlinear, with the greatest losses early in the mission. It is also evident that skeletal muscle atrophy and dysfunction are most prominent in the knee extensor (KE; −6% to −12%) and plantar flexor (PF; −6% to −24%) muscle groups [6].

The physiological mechanisms of disuse- or unloading-related skeletal muscle atrophy have been previously reviewed [7,8]. In brief, a change in skeletal muscle size is a reflection of the temporal rates of muscle protein synthesis and degradation. For instance, across a 24-h period, if the rate of muscle protein synthesis is greater than the rate of breakdown, net muscle protein balance will be positive and protein will be gained. In contrast, if the rate of muscle protein breakdown is greater than the net rate of muscle protein synthesis, the net balance will be negative and protein will be lost. Although debated, evidence suggests that unloading induced skeletal muscle atrophy in humans occurs primarily as a result of decreased basal muscle protein synthesis and a reduced synthetic response following feeding [7,9]. In-flight, variables such as participation in exercise countermeasures, age, gender, genetics, stress level, total energy intake, macronutrient composition, and preflight fitness level can influence the rates of muscle protein synthesis and breakdown; therefore, it is difficult to systematically determine the most important factor facilitating spaceflight-induced skeletal muscle atrophy, though the unloading itself is assumed to be the most potent factor.

To defend against skeletal muscle atrophy and physiological deconditioning on the ISS, crewmembers perform 2.5 h per day (including time for equipment setup and breakdown) of aerobic and/or resistance exercise; however, exercise prescription, exercise preference, and exercise adherence vary among crewmembers. The exercise equipment on the ISS, including a treadmill, a cycle ergometer, and the Advanced Resistive Exercise Device (ARED), have been engineered to operate in a weightless environment and are housed within vibration isolation systems to prevent damage to the ISS structure. During treadmill exercise, crewmembers must wear special harnesses that attach to the sides of the treadmill via bungee cords to maintain contact with the treadmill surface and to provide musculoskeletal loading during exercise. Crewmembers adjust the length of the bungee cords to set the pull-down load to a percentage (usually 60%–80%) of 1-G body mass. The cycle ergometer exercise operates similarly to an upright/recumbent bicycle and is used primarily for cardiovascular and aerobic conditioning. Resistance exercise in space is particularly challenging because the microgravity environment precludes the use of traditional free weights or weight stacks. However, ARED offers over 20 different resistance exercises including squat (SQT), heel raise, dead lift, bench press, and upright row [10]. The resistance is provided by vacuum cylinders that offer up to 273 kg of applied external load [11], and inline flywheels simulate 1-G inertia when the load changes direction during exercise. Since crewmembers are weightless in microgravity, the large loading capability is required because body mass must be added to the prescribed load in order to observe similar musculoskeletal forces to those on the ground. Thus, the current ISS exercise hardware allows for high-intensity exercise, enabling crewmembers to run up to 12 mph on the treadmill and train with heavy loads using ARED.

Although ARED and the other exercise devices provide excellent loading and resistance capabilities on the ISS, this specialized exercise hardware is expensive to build, launch, and maintain in an isolated spaceflight environment. Further, maintenance and repair must be performed by crewmembers, and spare parts for the complex devices may not be immediately available. Exercise hardware for future long-duration exploration missions may need to be even more robust, compact, and portable than the current devices. There may also be fewer modes of exercise available to crewmembers, and aerobic and resistance trainings may have to be performed on a single device. It is therefore important to investigate adjunct therapies that might enhance the effectiveness of aerobic and/or resistance exercise in the event that the exercise hardware itself poses limitations. Exercising at a low intensity with blood flow restriction (via inflation cuffs) has recently become a popular method of training in Japan [12]. The purpose of this review is to present an overview of BFR exercise training and discuss its potential usefulness as an adjunct exercise countermeasure for prolonged spaceflight.

### BFR exercise prescription and training

#### BFR resistance training

BFR resistance exercise training (also known as KAATSU when specific equipment is utilized [12]) combines low training intensity (approximately 20%–50% 1RM) with an external pressure cuff applied to the exercising limb [13-16]. BFR exercise protocols vary and are primarily influenced by cuff size, pressure, and the circumference (CIRC) of the limb being exercised [17]; however, most use three to five sets of exercise with 30- to 90-s rest periods [18]. Maintaining the cuff pressure (CP) during exercise and the rest interval also appears to be an important variable in order to increase the metabolic demand in both type I and type II muscle fibers [19]. As a result, the total number of repetitions performed in a training session can vary and is also determined by whether the first set of BFR exercise is to volitional fatigue [18] or a predetermined number of repetitions [20].

**Figure 1:**
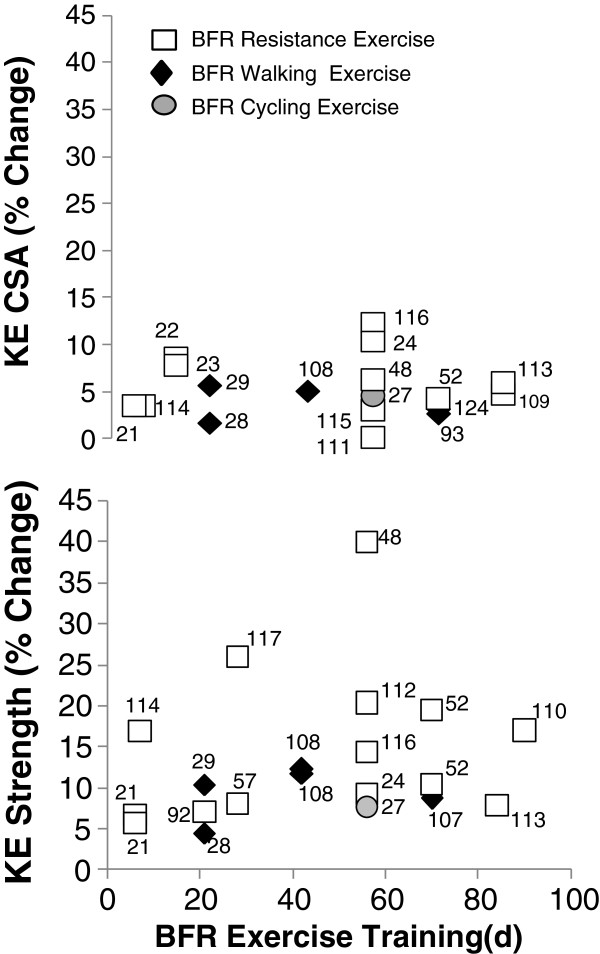
**Relative change (%) KE muscle size and strength with BFR exercise training.** Numbers in the figure correspond to reference citations [27,28,107-118].

Overall, BFR exercise training studies have ranged from 6 to 90 days in duration (Figure 1) and most show dramatic muscular adaptations. For example, Fujita et al. [21] reported 6.7% and 3% increases in KE strength and size, respectively, after 6 days of twice-daily BFR knee extension (KEx). Likewise, Yasuda et al. [22] demonstrated even larger improvements in 1RM SQT (14%) and quadriceps muscle size (7.8%) from 2 weeks of twice-daily BFR SQT exercise. Moreover, BFR SQT and leg curl exercise performed 6 days per week for 2 weeks resulted in approximately 17% and 22% increases in SQT and leg curl 1RM, respectively, and an 8.5% increase in thigh cross-sectional area (CSA) [23]. Studies implementing less frequent BFR resistance exercise sessions over longer training durations also show substantial muscular adaptations. Clark et al. (2011) observed an 8% increase in KE strength after 4 weeks of BFR exercise (3 days per week), while Takarada et al. [24] reported approximately 10% increases in KE strength and size over 8 weeks (2 days per week) of training. When studies are statistically combined, mean effect sizes ((post-mean − pre-mean)/pre-standard deviation and adjusted for sample size bias) [25] for muscle hypertrophy and strength for BFR resistance exercise are 0.39 and 0.58, respectively (compared to 0.01 and 0.000 for low-load training without cuff inflation) [15]. An important difference between high-load and BFR training is that increased muscle strength corresponds with muscle hypertrophy within the first 4 weeks of BFR exercise training, which is in contrast with the nervous system adaptations that result in enhanced muscle strength over the same duration of high-load resistance exercise training [26].

#### BFR aerobic training

Similar to BFR resistance training, the BFR aerobic exercise training studies couple low-intensity walking or cycling (approximately 20%–40% of maximal oxygen consumption, VO_2_max) with an external pressure cuff of approximately 200 mmHg applied to the upper legs [27-29]. Walk training studies showed that the metabolic cost of walking is approximately 3% higher and that heart rate (HR) is approximately 30 bpm higher during walking with BFR compared to normal walking at the same speed [29]. The training adaptations of 3-weeks of twice-daily BFR walking training at 20% of VO_2_max included an increase in leg press and leg curl 1RM of approximately 8% and an increase in upper leg CSA of approximately 6%. No changes in muscle size or strength were observed in the control group that performed the same walking protocol without BFR [28,29]. Similarly, 3 weeks of three times-per-week BFR cycling for 15 min at 40% VO_2_max resulted in increases in thigh CSA (3.4%), KEx strength (7.7%), and VO_2_max (6.4%). The control group cycled for 40 min at the same intensity and showed no change in muscle strength or aerobic fitness [27]. Traditionally, aerobic exercise is prescribed at approximately 75% of VO_2_max to elicit improvement in aerobic fitness. BFR aerobic exercise not only improves aerobic fitness at a low intensity, but also increases muscle strength and size, which are not usually observed following an aerobic training program.

### Mechanisms underlying BFR exercise training adaptations

Data from BFR exercise training studies demonstrate that this novel exercise approach is gaining scientific merit as a potential alternative to traditional resistance exercise, and a growing body of literature is emerging to suggest a potential to also improve aerobic fitness. There was a recent surge in publications describing the underlying mechanisms associated with BFR resistance training adaptations; however, the physiological mechanisms explaining the observed changes remain elusive.

#### Muscle metabolism, motor unit recruitment, and fiber activation

Evidence suggests that type II muscle fibers are recruited at a low load during BFR exercise [19]. A current hypothesis indicates that BFR resistance exercise causes type I muscle fibers to fatigue quickly due to low oxygen availability; hence, activation of type II muscle fibers and a greater reliance on anaerobic metabolism are required [14]. These events result in an accumulation of muscle metabolites that stimulate the production of systemic or local growth factors that initiate muscle protein transcription and translation [30-32]. A convincing evidence for type II muscle fiber activation was demonstrated by Krustrup et al. [19] who showed that phosphocreatine concentrations in both slow and fast twitch fibers following BFR resistance exercise were reduced to an equivalent concentration as compared to high-intensity resistance exercise. However, fast twitch fiber recruitment evaluated by inorganic phosphate splitting occurred in only 31% of participants performing one set of BFR resistance exercise compared to 70% of subjects performing one set of high-load exercise [32]. When multiple sets of BFR exercise were performed with continued BFR during the rest intervals, inorganic phosphate splitting was similar to multiple sets of high-load resistance exercise [33]. Therefore, the overall effort and threshold of fatigue reached during a session may facilitate the acute training response [34]. Further evidence for insufficient oxygen availability and anaerobic type II fiber activation exists in studies reporting enhanced muscle biopsy lactate [35], blood lactate (La) [20], and decreased pH [20] following BFR exercise compared to exercise at the same load without external CP [30,31].

#### Systemic hormonal response

Anabolic and catabolic hormonal responses have been frequently evaluated following acute and chronic BFR resistance exercise (Table 1). It is hypothesized that accumulation of metabolic by-products and/or the hypoxia-induced stimulation of afferent nerve fibers results in an increase in secretion of the growth hormone (GH) and GH-releasing hormone [36]. Takarada et al. [30] reported that circulating GH concentrations following an acute bout of BFR exercise were 290 times greater than the baseline; however, Pierce et al. [37] showed a lower but still physiologically significant ninefold increase in GH concentration using a similar protocol. A corresponding increase in the circulating insulin-like growth factor-1 (IGF1) has been observed during BFR KEx exercise (20% 1RM, four sets to exhaustion, 160–180 mmHg) and at 10–30 min post-exercise [36]. In contrast, the circulating IGF1 concentration was not increased up to 180 min following an acute bout of BFR KEx exercise (20% 1RM, 200 mmHg, four sets, 75 total repetitions, 30-s rest periods) [20]. It is also argued that the increase in IGF1 observed in some studies could be related to a hemoconcentration as a result of plasma volume (PV) changes following BFR resistance exercise [13]. Following a training period, there was a report of a progressive increase in circulating IGF1 at rest following 2 weeks of twice-daily BFR SQT and leg curl exercise (20% 1RM, three sets, 15 repetitions/set, 30-s rest periods) [23]. Therefore, the overall relationship between BFR resistance exercise and the GH-IGF1 axis remains controversial.

**Table 1:** Systemic biomarkers and hormonal responses to an acute bout of BFR exercise

**Reference citation**	**Age(year)**^**a**^	**Exercise(s)**	**Intensity**	**Cuff width**	**CP(mmHg)**	**Significant increase(*p* < 0.05)**	**No change(*p* > 0.05)**
[39]	21	EF,PFx	30% 1RM	NR	20↓ SBP	La, GH	T, Cort
[20]	32	KEx	20% 1RM	NR	200	La, GH, Cort	IGF1, T
[40]	26	EF,EE,KEx,KF	30% 1RM	30 × 450 mm	130–200	La, Cort, NorEpi, GH, T	
[29]	21	Walking	50 m/min, 2 min	200 × NR mm	200	GH	Cort
[30]	20–22	KEx	20% 1RM	33 × 800 mm	214	La, NorEpi, GH, IL6	CK, LP
[119]	25–40	EF	30%–50% 1RM	90 × 700 mm	0–100	La	
[120]	24–28	EF,EE,SQT,KF	20% 1RM	30 × 45 mm	190–230	La, NorEpi, GH, Hemat	Na, K,
[121]	20–22	KEx	30% 1RM	NR	200	La, NorEpi, GH	
[37]	22	KEx	20% MVC	NR	280	GH	
[21]	22	KEx	20% 1RM	NR	160–220		CK, Myo, IL6
[52]	21	EF,KEx,KF	30% 1RM	40 × 1,750 mm	>160	GH, NorEpi	T
[42]	70	KEx	20% 1RM	NR	200	La, Cort, GH	Glu
[36]	34	KEx	20% 1RM	33 × 880 mm	160–180	La, NorEpi, GH, IGF1, VEGF	Ghrl

^a^Age expressed as a mean, or if not available, as a range; *CP* cuff pressure, *NR* not reported, *EF* elbow flexion, *PFx* plantar flexion, *KEx* knee extension, *EE* elbow extension, *SQT* squat, *Myo* myoglobin, *La* blood lactate, *NorEpi* norepinephrine, *IL6* interleukin-6, *LP* lipid peroxidase, *CK* creatine kinase, *pH* blood pH, *Cort* cortisol, *IGF1* circulating insulin-like growth factor 1, *T* testosterone, *Na* sodium, *K* potassium, *Myo* myoglobin, *Glu* glucose, *Ghrl* ghrelin, *VEGF* vascular endothelial growth factor.

The influence of acute and chronic BFR exercise on other anabolic hormones such as testosterone (T) is also unclear [38]. For example, neither Reeves et al. [39] nor Fujita et al. [20] observed changes in total or free following arm or leg BFR resistance exercise, respectively. In contrast, Madarame et al. [40] reported a post-exercise elevation in total T following three sets of BFR KEx and flexion. Chronic BFR walking [29] or resistance exercise [41] has also failed to show resting changes in T. Worthy of note, studies do show that circulating cortisol (Cort) is elevated following BFR and high-load resistance exercise [20,40,42], which suggests a similar stress response. However, circulating Cort concentration is primarily associated with catabolism and muscle protein breakdown [43]. Recently, the overall association between the acute systemic hormonal response to resistance exercise and muscle hypertrophy has been questioned. West et al. [44] reported no additional rise in muscle protein synthesis or the phosphorylation of signaling proteins following resistance exercise during elevated systemic concentrations of T, GH, and IGF1 compared to low systemic concentrations of the same anabolic hormones. Hence, local factors may provide greater stimuli to induce BFR resistance exercise adaptations.

#### Gene expression and cell signaling

An acute bout of high-load resistance exercise elicits anabolic and catabolic responses that are altered in a complex temporal manner to achieve muscle hypertrophy. Briefly, muscle protein synthesis and myogenic gene transcripts (e.g., myogenin) are upregulated within 2 h post-exercise and peak approximately 8 h post-exercise, whereas proteolytic ligases (muscle-specific RING finger protein-1 (MuRF-1), Atrogin-1) are upregulated 1–4 h after exercise and downregulated within 8 h of exercise termination [45]. An approximate threefold increase in the phosphorylation of ribosomal protein S6 kinase beta-1 (S6K1), a downstream component of the mammalian target of rapamycin (mTOR) signaling pathway and regulator of translation initiation and elongation, and a 46% increase in the fractional synthesis rate (a measure of muscle protein synthesis) have been previously shown following BFR resistance exercise [20]. mRNA expression of genes that regulate satellite cell activity (mechano-growth factor, IGF1 receptor, myogenin, MyoD), cell size (myostatin), and protein turnover (MuRF1, mTOR, S6K1) were not different between BFR and low-load exercise up to 3 h post-exercise [46]. At 8 h post-BFR or low-load exercise, myogenic gene transcripts (IGF1, MyoD, and myogenin) were not different from the baseline; however, proteolytic transcripts (Atrogin-1, MuRF-1, and Forkhead box O3 (FOXO3A)) were downregulated twofold from baseline in the BFR group only [47]. Following 8 weeks of BFR resistance training, myostatin gene expression was downregulated to a similar extent compared to high-load training [48], with a trend (*p* = 0.06) toward decreased activin IIb (myostatin receptor). There were also elevations in genes associated with myostatin function (growth and differentiation factor-associated serum protein-1) and signaling (SMAD family) [48]. Overall, the molecular pathways that regulate BFR resistance exercise-induced muscle hypertrophy have not been extensively studied. Given that muscle hypertrophy results from a positive net muscle protein balance (synthesis > breakdown) across a training period, greater examination of molecular events is needed to better understand how BFR resistance exercise stimulates muscle growth.

### The limitations and safety of BFR exercise

#### BFR exercise limitations

The application of BFR exercise appears to be limited to peripheral muscle groups; thus, core, back, and neck muscles cannot be specifically targeted using this methodology. Higher perceptual ratings of perceived exertion and pain during the rest intervals of sets have also been reported, which could limit the application of this training methodology [49]. The pressure applied to the blood vessel during BFR exercise is likely the root cause of discomfort and is associated with cuff width [17] and the layer of soft tissue situated between the cuff and the vessel [50]. The cuff sizes most frequently used in research studies are either narrow (approximately 5 cm) or wide (approximately 13 cm). From the data, it appears that lower cuff pressures (90–120 mmHg) are required to occlude venous blood flow when the wider cuffs are used compared to the narrow cuffs (pressures, 160–180 mmHg [51]. However, the narrow cuffs and associated pressures have repeatedly been shown to cause improvements in muscle strength and size when combined with low-load resistance exercise [52]. Exercise training adaptations have been observed with wider cuffs with the same [53,54] or lower cuff pressures [55]; however, BFR exercise performed at supra-systolic blood pressure (SBP) with wider cuffs may restrict blood flow to a level that reduces exercise volume and increases discomfort compared to narrower cuffs [56]. Therefore, cuff width is an important variable for determining BFR exercise prescription and may be a limiting factor if not taken into consideration with CP.

#### Hemostasis

The potential for blood coagulation and venous thrombus following blood pooling is the most frequently discussed (hypothesized) risk, and therefore, it has been comprehensively evaluated in multiple acute studies (Table 2). Most recently, Clark et al. [57] observed an increase in tissue plasminogen activator (tPA), a fibrinolytic protein that catalyzes the conversion of plasminogen to plasmin, immediately following a single bout of BFR exercise. This finding is consistent with those of Nakajima et al. [58] who reported that an acute bout of BFR exercise increased tPA antigen without altering plasminogen activator inhibitor-1 (PAI-1) or D-dimer (D-d). Together, these results suggest that BFR exercise may acutely increase fibrinolytic activity, thus reducing the risk for blood coagulation.

**Table 2:** Hemostasis markers with acute BFR resistance exercise

**Reference citation**	**Exercise**	**Intensity**	**Sets(total repetitions)**	**CP(mmHg)**	**Cuff width(mm)**	**Significant increase(*p* < 0.05)**	**No significant change(*p* > 0.05)**
[57]	KEx	30% 1RM	3 (24–36)	1.3 *×SBP	80 × 830	tPA	Fib, D-d, PTF
[20]	KEx	20% 1RM	4 (75)	200	NR		TAT, PTF 1,2, D-d
[42]	KEx	20% 1RM	4 (75)	200	NR		D-d
[122]	Leg press	30% 1RM	4 (75)	150–160	65 × 650		PTF, TAT, D-d
[58]	Leg press	30% 1RM	4 (75)	172.5	NR	tPA	D-d, Fib, PAI-1

*SBP* systolic blood pressure, *NR* not reported, *TAT* thrombin-anithrombin complex, *tPA* tissue plasminogen activator, *D*-*d* D-dimer, *Fib* fibrinogen, *PAI*-*1* plasminogen activator inhibitor-1, *PTF* prothrombin fragment 1,2.

#### Acute cardiovascular stress

The acute cardiovascular responses to BFR exercise training are important to consider because this type of exercise could provide the greatest benefit for individuals with a variety of health risks that preclude them from performing traditional resistance exercise. Although most BFR literature focus on muscular effects, it is important to note that resistance and aerobic BFR exercises cause increases in HR and blood pressure that are greater than those observed with exercise performed at a similar intensity without BFR [29,59]. The increase in HR is an important aspect of BFR exercise because it allows cardiac output (CO) to be maintained, despite a decrease in venous return due to the CP [36]. Since most BFR studies are conducted in healthy subjects, it is important to evaluate the cardiovascular response to BFR in individuals presenting with cardiovascular disease risk factors in a controlled setting.

#### Muscle damage and reperfusion

Maintaining CP during the between-set rest interval is one essential feature of the BFR exercise prescription. As a result, higher levels of muscle soreness and perceived exertion have been reported during and/or following BFR-restricted exercise compared to the same low-intensity exercise performed without CP [60,61]. Delayed onset muscle soreness is a common occurrence following eccentric muscle actions during high-load resistance exercise [62]. In contrast, it appears that concentric muscle actions compared to eccentric muscle actions result in greater muscle soreness following BFR resistance exercise [60]. It is unclear why this is the case; however, eccentric-induced muscle soreness is typically associated with mechanical stress, which may be attenuated with BFR exercise given the very low training loads. Systemic physiological markers of BFR resistance exercise-induced muscle damage are conflicting. Although neither creatine kinase nor myoglobin were elevated following two BFR resistance exercise bouts [20,30], one case of rhabdomyolysis has been reported in the literature [63].

In addition to the potential for muscle damage with BFR training, there is a hypothetical risk for microvascular dysfunction as a consequence of the reperfusion that occurs when blood flow is restored after a period of restriction or ischemia [64]. During reperfusion, there is an acute release of inflammatory molecules, clotting factors, and reactive oxidative species that impair microvascular function [65]. Further, nitric oxide bioavailability (a mediator of vasodilation) decreases when blood flow is restored, causing impaired arterial vasodilation and increased sheer stress. Repeated reperfusion injury can eventually cause a wound that influences endothelial function. Renzi et al. [64] have shown a significant reduction in flow-mediated vasodilation 20 min after BFR walking exercise, suggesting the potential for endothelial dysfunction. Recent evidence also suggests increased sarcolemma permeability (evidenced by staining of tetranectin) following BFR exercise, which may be caused by cell damage from increased production of reactive oxygen species [66]. Furthermore, although not statistically significant, Goldfarb et al. [67] showed that both protein carbonyls and glutathione ratios (systemic indicators of oxidative stress) were almost doubled following BFR resistance exercise in seven male subjects. Given that the time course of reactive oxygen species generation was limited to 15 min post-exercise, it is essential that future acute studies have a sufficiently high number of subjects and extended time courses.

Overall, BFR exercise training encompasses a variety of new variables (cuff width, CP, cuff inflation duration) for exercise prescription, and understanding these interactions in terms of safety is complex [68]. To date, the most comprehensive data set on side effects from BFR exercise training was established using survey methodology. The most reported incidents from approximately 13,000 people participating in KAATSU training were as follows: bruising (13.1%), numbness (1.3%), cerebral anemia (0.3%), cold feeling (0.1%), pulmonary embolism (0.01%), rhabdomyolysis (0.01%), deterioration of ischemic heart disease (0.02%), and venous thrombus (0.06%) [69].

### BFR exercise in space—potential applications

Crewmembers commonly experience losses in aerobic capacity and muscular strength following long-duration spaceflight. Crewmembers that perform daily moderate- to high-intensity exercise throughout the mission duration generally return in considerably better condition than their counterparts that engage in little or low-intensity activity. Exercise with BFR may provide a means to perform resistance exercise with a low load or perform aerobic exercise at a slower walking speed or lower pedaling resistance in the event of ISS exercise hardware failure or in future exploration missions with less robust exercise hardware.

#### Muscle size, strength, and endurance

Cook et al. [70] recently showed that KE CSA was maintained (−1%) in a group that performed BFR resistance exercise over a 30-day period of lower-limb unloading compared to a non-exercise control group (−7.5%). Similarly, maximal voluntary contraction (MVC) was also preserved in the BFR resistance exercise group (−2%), while the control group exhibited a 15.6% decline. Surprisingly, the BFR group also had a 28% increase in submaximal (approximately 40% MVC) muscular endurance compared to a 24% decline in controls. These results suggest that BFR resistance exercise may be an effective exercise countermeasure to prevent losses in KE size and strength while simultaneously improving muscular endurance [70].

The plantar flexors are another major muscle group susceptible to atrophy during unloading with the greatest plasticity evident in the soleus. For example, following 6 months on the ISS, soleus muscle size declined by 18% [71-73] and was accompanied by a decrement in PF peak torque of 20%–29% across the velocity spectrum [73]. Even more concerning is that these results were evident despite the performance of resistance and aerobic exercise countermeasures [73]. It is hypothesized that the improved loading capability (273 kg) of ARED onboard the ISS will mitigate future changes in soleus muscle size and attenuate atrophy of other muscle groups. However, the soleus muscle is best isolated when the knees are flexed, which reduces the contribution of the gastrocnemius to the muscle action [74]. Currently, this motion is difficult to reproduce using ARED because movements are primarily performed in the standing position. Another problematic aspect of targeting the soleus is a limited range of motion. As loads are increased during plantar flexion exercise, the range of motion can decrease substantially. Thus, because BFR resistance exercise is performed using low training loads, it may be possible to exercise throughout a crewmembers' full anatomic range of motion to more effectively target the soleus. Although BFR resistance exercise has not yet been performed in an unloading analog, a recent ground-based study showed a 30% increase in PF MVC following 4 weeks of training; these findings help to substantiate its potential application as a countermeasure to unloading [55].

#### Orthostatic challenge

BFR exercise is also a potential countermeasure to orthostatic intolerance [75-77], a condition reported to occur in up to 30% of astronauts returning from brief space shuttle flights of 4–10 days [78] and in 80% of astronauts following long-duration missions [79]. During exposure to microgravity, blood volume (BV) shifts from the capacitance vessels of the lower body to those in the face and head. Upon return to 1-G, there is excessive pooling in the lower limbs, resulting in orthostatic hypotension and syncope [80]. Research has shown that elastic cuffs worn on the upper thighs during flight help to maintain central and peripheral hemodynamics and mitigate post-flight orthostatic intolerance [81]. However, the ability of these cuffs to completely mitigate cardiovascular deconditioning during spaceflight has not been definitively determined [82]. It is hypothesized that the most effective in-flight countermeasure would be a gravity-like stimulus, such as lower body negative pressure (LBNP). Exercise with LBNP of 1.0 to 1.2 times body weight during −6° head-down-tilt (−6HDT) bed rest has been shown to maintain upright exercise capacity [83].

BFR exercise elicits several features that are similar to LBNP including lower extremity blood pooling, decreased venous return, and increased autonomic activation [75]. Nakajima et al. [75] showed that restriction of blood flow reproduces the effects of standing on HR, stroke volume (SV), and norepinephrine release, thus simulating a gravity-like stress during short-duration −6HDT bed rest. Furthermore, Kubota et al. [76] demonstrated that BFR resistance exercise during short-duration −6HDT bed rest elicits hemodynamic and neurohumoral responses that approximate a gravity-specific stress on the cardiovascular system. In the before-mentioned study, subjects participated in 24 h of −6HDT bed rest resulting in 4.4% and 7.8% losses of BV and PV, respectively. Subjects performed BFR leg press resistance exercise (30% 1RM, four sets, 30/15/15/15 repetitions, 1-min rest intervals, 65 × 650-mm cuff width, 150–160 mmHg CP), while remaining at −6HDT. SV was significantly reduced during BFR resistance exercise and was similar to the measurement obtained when standing. These results suggest that frequent applications of BFR exercise during microgravity may provide a stimulus to the cardiovascular system that simulates 1-G, which may reduce post-flight orthostatic intolerance. Future investigations should examine the impact of BFR exercise during long-duration bed rest on the physiological responses to orthostatic stress.

### BFR exercise in space—unanswered questions

To date, the majority of evidence for the application of BFR in space stems from research exploring skeletal muscle physiology during acute or chronic exercise studies. Few BFR studies have focused on the acute effects or training adaptations on cardiovascular or skeletal health. Since exposure to microgravity compromises human physiology in a variety of ways, there is a clear need to expand the scope of BFR research to include other physiological systems.

#### Cardiovascular health

Cardiac mass decreases to levels that are well below normal in conditions of weightlessness and simulated weightlessness. Perhonen et al. [84] demonstrated that left ventricular mass (measured by magnetic resonance imaging) decreased by 15% during prolonged supine bed rest and by 12% after short-duration spaceflight. It appears as though disuse-induced cardiac atrophy does not influence systolic function [84]; however, one of the most important clinical consequences of cardiac atrophy may be its influence on diastolic function. Invasive studies of cardiac performance before and after 2 weeks of −6HDT bed rest have shown that there is a leftward shift in the diastolic pressure-volume curve after bed rest, resulting in a smaller left ventricular end-diastolic volume for a given filling pressure [85]. Because the effects of BFR on cardiac morphology and function have not yet been investigated, it is unclear if this form of exercise could provide the stimulus required to completely prevent cardiac deconditioning. The novelty of BFR appears to be the unique combination of venous BV pooling and restricted arterial blood inflow, resulting in a decreased SV and increased HR, while maintaining CO [36]. Consequently, in contrast to traditional aerobic exercise [86], eccentric loading of the heart and prevention of cardiac atrophy may not occur during BFR exercise.

Previous studies have established that deconditioning leads to detrimental vascular changes such as endothelial dysfunction, decreased arterial compliance, and atherosclerosis [87,88]. Hesse et al. [89] found that 13 days of bed rest impaired endothelium-dependent arterial relaxation in healthy men, while Tuday and colleagues [88] reported that spaceflight significantly reduced vessel compliance [88]. Alternatively, cross-sectional studies using middle-aged and older adults have found that regular aerobic exercise improves arterial compliance [90,91]. As previously mentioned, although BFR exercise appears to acutely decrease endothelial function [64], the chronic effects of BFR exercise remain equivocal. Kim et al. [92] found that arterial compliance of the large and small arteries was not affected by 4 weeks of BFR resistance training. Conversely, Ozaki et al. [93] reported that carotid arterial compliance was improved by 10 weeks of BFR walk training in elderly subjects. Given that reduced endothelial function and arterial compliance are early markers of atherosclerosis [94], surrogate markers of cardiovascular function [95], and predictors of future cardiovascular complications [96,97], further examination of both the acute and chronic effects of BFR exercise on vascular health is needed.

#### Skeletal health

In addition to the cardiovascular consequences associated with prolonged spaceflight, bone health is a primary concern. Bone demineralization occurs predominately in the long bones of the lower limbs, with maximal bone loss occurring in the calcaneus and the hip [98]. Skeletal unloading in astronauts can result in losses of 1%–2% per month in bone mineral density [99,100]. On the Mir year-long mission, bone measurements of astronauts showed a 10% reduction of the lumbar vertebrae [101]. Despite having a lower mechanical load during training, there is some evidence in ambulatory subjects that BFR resistance training may be beneficial to bone. For instance, after an acute bout of BFR resistance exercise in young men, there was a significant reduction in bone resorption as evidenced by serum N-terminal cross-linking telopeptide of type I collagen [102]. Six weeks of BFR resistance exercise training in older men also showed a 21% increase in serum bone-specific alkaline phosphatase, a marker of bone formation [102]. Although the mechanism for how low-load BFR resistance exercise improves bone parameters has not been clearly established, Loenneke et al. (2012) indicate that BFR exercise may induce physiological responses as a result of interstitial fluid flow-induced sheer stress within the osteocyte membrane [103] and/or the activation of vascular endothelial growth factor via the hypoxia inducible transcription factor pathway [104]. Further work is needed to determine how BFR training could protect bone health during prolonged unloading.

### Perspectives

A BFR exercise training device and Russian Braslet cuffs are currently onboard the ISS. To our knowledge, these devices have not been used with exercise by ISS crewmembers. However, BFR exercise may have already been inadvertently performed by US astronauts on Skylab. In a recent historical account, it was noted that during the first few days of the mission, astronauts had significant problems adhering to the exercise workloads prescribed on the cycle ergometer [105]. At low intensities, no issues were observed; however, at high intensities, workloads could not be reached and the astronauts described significant leg fatigue and discomfort [105]. The root cause was the combination of a padded waist belt and shoulder harness that restricted circulation to the legs as training loads increased. A solution to the problem was eventually implemented, but it is plausible that the restricted circulation these crewmembers experienced was similar to the BFR cycling methods that have been recently evaluated by researchers [27].

Overall, it is unlikely that BFR exercise will be used as a standalone exercise countermeasure onboard the ISS given the significant presence of ARED among other more traditional exercise devices. However, in future space exploration missions beyond the ISS, both aerobic and resistance BFR exercise trainings may be given some consideration depending on the vehicle capacity and the capability of onboard exercise devices. Combining low-load BFR resistance exercise with the current moderate- to high-load could help attenuate skeletal muscle atrophy without excessive loading of the shoulders, lower back, and/or joints. Specifically targeting the plantar flexors with BFR resistance exercise training may be an alternative method to prevent soleus atrophy and dysfunction. Research also suggests that BFR exercise may simulate the cardiovascular response to standing in 1-G, which could potentially reduce orthostatic intolerance upon return to earth. However, few studies have evaluated BFR exercise using a chronic ground-based unloading analog (Table 3), and only two acute studies have used −6HDT bed rest, which is the analog that reflects the fluid shift observed during spaceflight.

**Table 3:** Restriction of blood flow and BFR exercise while supine or using musculoskeletal unloading models

**Reference citations**	**Method**(**s**)	**Highlighted outcome(s)**	**Limitation(s)**
[75]	KAATSU (65 × 650-mm cuff, 50–100 mmHg, no muscle contractions, 10 min) following 24-h −6HDT bed rest	24 h of −6HDT bed rest resulted in ↓body mass,↓BV, ↓PV, and ↓IVCd; 10 min of 50 mmHg, KAATSU: ↑HR, ↓SV, ↓CO, ↓IVCd, ↔Hct, ↔Hb, ↔BV, and ↔PV. Authors suggest that KAATSU reproduces the effect of a gravity-like stress during simulated weightlessness	One subject developed neurocirculatory presyncope 5 min after 100 mmHg KAATSU. There were no symptoms in the remaining seven subjects
[76]	KAATSU (65 × 650-mm cuff, 150–160 mmHg) with −6HDT leg press resistance exercise (30% 1RM, four sets, repetitons: 30/15/15/15, 1-min rest between sets) following 24-h −6HDT bed rest	24 h of −6DHT bed rest: ↓body mass, ↓BV, ↓PV, and ↑Hct. KAATSU + −6DHT leg press resistance exercise:↑HR, ↑BP, ↓SV, and ↑CO. Authors suggest that KAATSU with leg press exercise mimics the exercise hemodynamic response to exercise in 1-G	Potential conflict of interest between the KAATSU device and the journal publishing the research study
[77]	Supine with KAATSU (60 × 605 mm, 200 mmHg, no muscle contractions vs. standing)	Supine with KAATSU: ↓SV, ↑HR, ↑TPR, and ↓CO. Authors suggest that KAATSU induced hemodynamics similar to standing	Case study: potential conflict of interest between the KAATSU device and the journal publishing the research study. Fluid shift stimuli are not introduced
[123]	Supine with KAATSU (60 × 605 mm, 50–250 mmHg, no muscle contractions vs. standing)	Supine with KAATSU: ↑FVd, ↓FBf, ↓IVCd, ↓LVDd, ↓CO, ↑HR, and ↑TPR. Authors suggest that KAATSU induced hemodynamics similar to standing	Fluid shift stimuli are not introduced
[70]	BFR (60 × 830 mm, 150 mmHg) KEx resistance exercise (20% MVC, three sets, repetitons to fatigue 1.5-min rest between sets) during 30 days of unloading via ULLS	Following 30 days of ULLS: ↔KE CSA, ↑KE endurance, ↓PF CSA, ↓PF MVC, ↔IGF1, and ↔IGFBP3. Authors suggest that BFR exercise is effective in maintaining muscle size and strength and improving muscular endurance during unloading	Fluid shift stimuli are not introduced. ULLS model may not be appropriate for systemic blood markers
[124]	Restriction of blood flow (77 × 770 mm, 200 mmHg, five sets, 5-min bouts, 3-min rest between sets during 14 days of cast immobilization	Restriction of blood flow: ↔KE MVC, ↔PF CON60,↔leg/thigh CIRC, and ↔GH. Authors suggest that restriction of blood flow to the lower extremity prevents disuse muscular weakness	Fluid shift stimuli are not introduced. Cast immobilization model differs from spaceflight musculoskeletal unloading due to joint mobility

*BFR* blood flow-restricted, *BV* blood volume, *PV* plasma volume, *IVCd* inferior vena cava diameter, *HR* heart rate, *SV* stroke volume, *CO* cardiac output, *Hct* hematocrit, *Hb* hemoglobin, *TPR* total peripheral resistance, *FVd* femoral vein diameter, *FBf* femoral arterial blood flow, *LVDd* left ventricle end-diastolic dimension, *KE* knee extensor, *CSA* cross-sectional area, *CIRC* circumference, *PF* plantar flexor, *CON60* concentric 60° sec^−1^, *MVC* maximal voluntary contraction, *IGF1* circulating insulin-like growth factor, *IGFBP3* circulating insulin-like growth factor binding protein-3, *GH* growth hormone, *1RM* one repetition maximum, *6HDT* −6° head-down-tilt bed rest, *ULLS* unilateral lower limb suspension, ↓ decreased, ↑ increased, ↔ no change.

Future work should evaluate the safety of exercise prescription and determine the influence of new programming variables (e.g., leg CIRC, adipose tissue thickness, CP, and cuff width) on human physiology. Once acute exercise prescription is understood, BFR exercise should be evaluated across multiple physiological systems using long duration −6HDT bed rest. Measuring cardiovascular and skeletal outcomes in addition to skeletal muscle parameters during prolonged unloading is essential to enhance our understanding of the effects of this novel method of exercise. Furthermore, because BFR exercise training has implications for sarcopenia and other muscle wasting conditions, a mechanistic understanding of the cellular pathways resulting in muscle growth would be beneficial [106].

## Conclusion

Researchers should be commended for their pioneering efforts in understanding the mechanistic and adaptive responses to BFR exercise. Both BFR resistance and aerobic exercise prescription appear to be in early stages of development relative to traditional resistance and aerobic training. Currently, low-load BFR resistance exercise increases muscle size and strength in ambulatory participants and attenuates muscle atrophy and strength loss during periods of unloading. Low-intensity BFR aerobic exercise while ambulatory enhances muscle size and strength and simultaneously increases aerobic fitness. As the science behind BFR exercise training matures in the future, it is evident that this type of training could be applicable as an adjunct countermeasure to combat musculoskeletal and cardiovascular dysfunctions during missions beyond low-earth orbit.

## Abbreviations

1RM: One repetition maximum; −6HDT: −6° head-down-tilt; ARED: Advanced resistive exercise device; BFR: Blood flow restricted; BV: Blood volume; CIRC: Circumference; CK: Creatine kinase; CO: Cardiac output; CON60: Concentric 60° sec^−1^; Cort: Cortisol; CP: Cuff pressure; CSA: Cross-sectional area; D-d: D-dimer; EE: Elbow extension; EF: Elbow flexion; FBf: Femoral arterial blood flow; Fib: Fibrinogen; FOXO3A: Forkhead box O3; FVd: Femoral vein diameter; Ghrl: Ghrelin; Glu: Glucose; GH: Growth hormone; Hb: Hemoglobin; Hct: Hematocrit; HR: Heart rate; IGF1: Insulin-like growth factor-1; IGFBP3: Circulating insulin-like growth factor binding protein-3; IL6: Interleukin-6; ISS: International Space Station; IVCd: Inferior vena cava diameter; K: Potassium; KE: Knee extensor; KEx: Knee extension; La: Blood lactate; LBNP: Lower body negative pressure; LP: Lipid peroxidase; LVDd: Left ventricle end-diastolic dimension; mTOR: Mammalian target of rapamycin; MuRF-1: Muscle-specific RING finger protein-1; MVC: Maximal voluntary contraction; Myo: Myoglobin; Na: Sodium; NorEpi: Norepinephrine; NR: Not reported; PAI-1: Plasminogen activator inhibitor-1; PF: Plantar flexor; PFx: Plantar flexion; pH: Blood pH; PTF: Prothrombin fragment 1,2; PV: Plasma volume; S6K1: Ribosomal protein S6 kinase beta-1; SBP: Systolic blood pressure; SQT: Squat; SV: Stroke volume; T: Testosterone; TAT: Thrombin-anithrombin complex; tPA: Tissue plasminogen activator; TPR: Total peripheral resistance; ULLS: Unilateral lower limb suspension; VEGF: Vascular endothelial growth factor.

## Competing interests

The authors declare that they have no competing interests.

## Authors’ contributions

KH outlined a draft of the proposal and synthesized contributions from all authors. KH, ME, JS, and LPS wrote, revised, and edited the manuscript. All authors read, edited, and approved the final version of the manuscript.
